# Supplementary figures and images for: Normal embryonic development and neonatal digit regeneration in mice overexpressing a stem cell factor, Sall4
Source: PLoS One. 2022 Apr 28;17(4):e0267273. doi: 10.1371/journal.pone.0267273 (PMC9049339; doi:10.1371/journal.pone.0267273)

Fig 2I (SALL4)

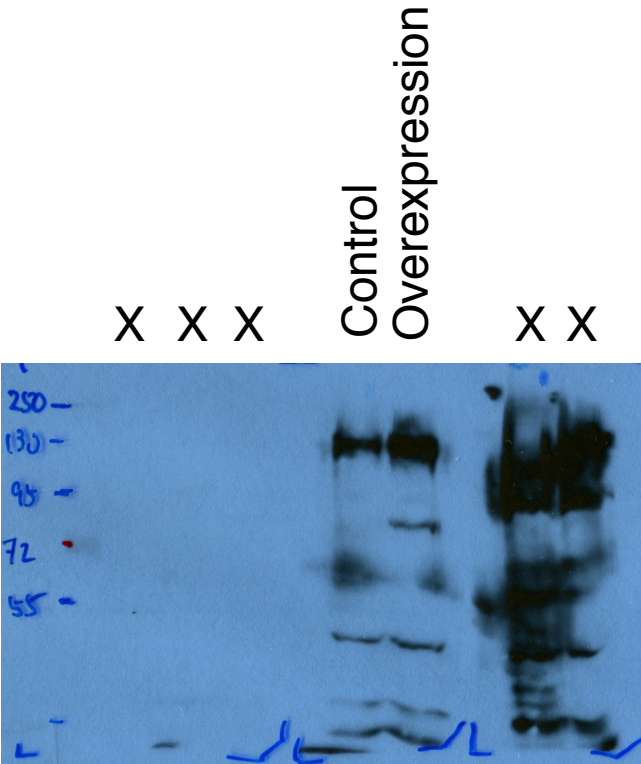

Fig 2I (GAPDH)

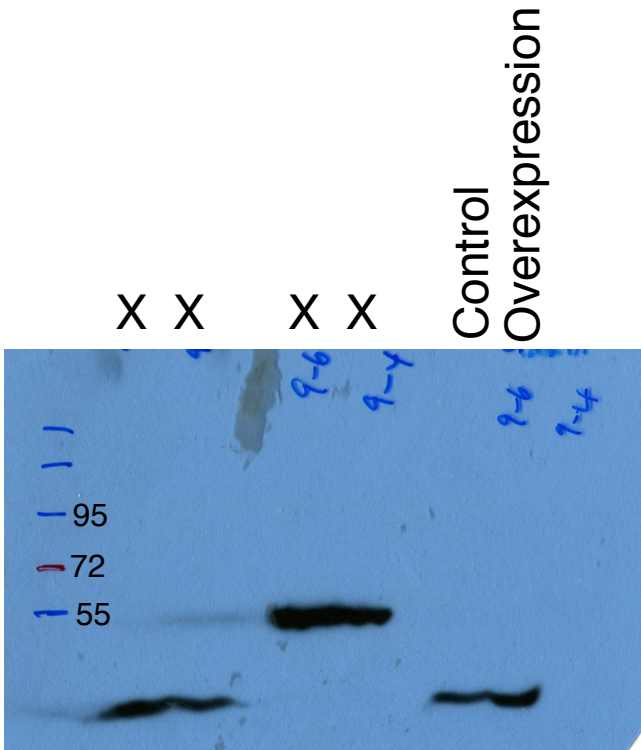

Fig 5A (SALL4)

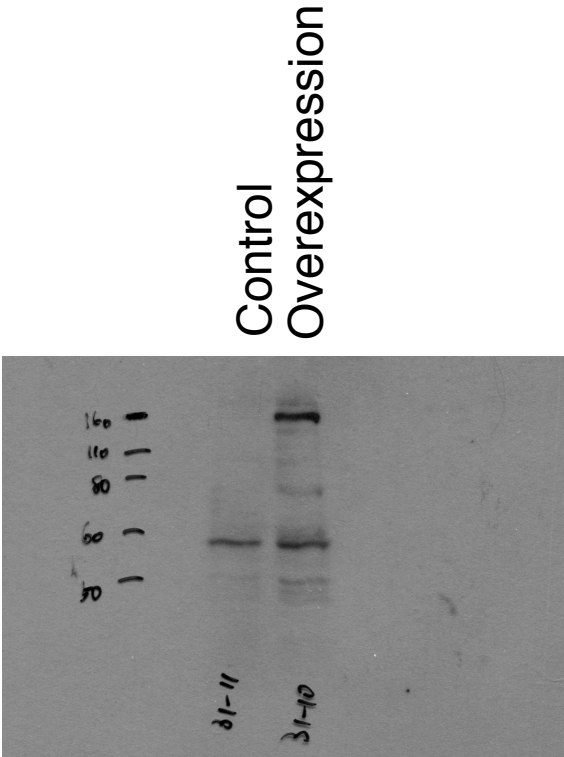

Fig 5A (GAPDH)

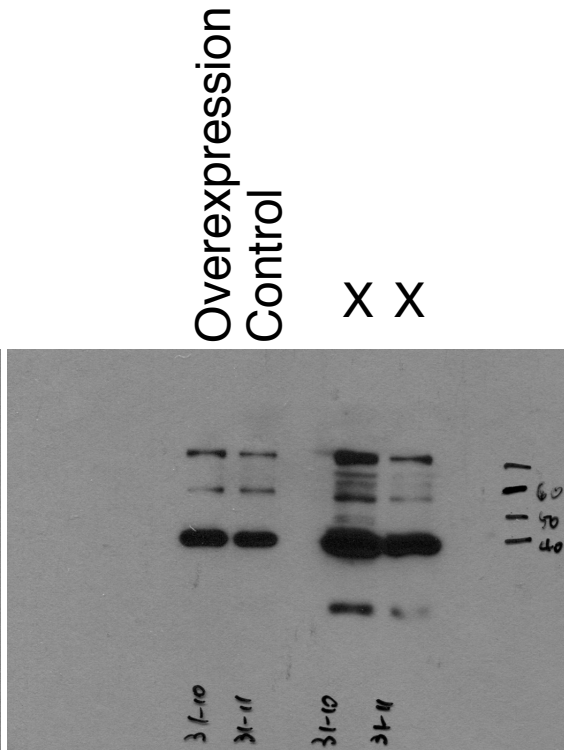

Supplement: S1 Raw images — (PDF) [file pone.0267273.s001.pdf]
